# Association of State Stay-at-Home Orders and State-Level African American Population With COVID-19 Case Rates

**DOI:** 10.1001/jamanetworkopen.2020.26010

**Published:** 2020-10-23

**Authors:** Sangeetha Padalabalanarayanan, Vidya Sagar Hanumanthu, Bisakha Sen

**Affiliations:** 1 Department of Health Services Administration, University of Alabama at Birmingham, Birmingham; 2 University of Alabama at Birmingham School of Health Professions, Birmingham; 3 Division of Clinical Immunology and Rheumatology, University of Alabama at Birmingham, Birmingham; 4 Department of Health Care Organization and Policy, University of Alabama at Birmingham, Birmingham

## Abstract

**Importance:**

To cope with the continuing coronavirus disease 2019 (COVID-19) pandemic, state and local officials need information on the effectiveness of policies aimed at curbing disease spread, as well as state-specific characteristics, like the racial mix, associated with increased risks related to the disease.

**Objective:**

To investigate whether state-imposed stay-at-home orders (SAHOs) and the proportion of African American population in a state were associated with the state-level COVID-19 cases.

**Design, Setting, and Participants:**

This cross-sectional study used daily, state-level data on COVID-19 cases, tests, and fatalities from the COVID Tracking Project. Data from March 1 to May 4, 2020, for all states (except Washington state) as well as the District of Columbia were used.

**Exposures:**

The key exposure variables were state-level SAHO (1 if in place, 0 otherwise), and proportion of state population who are African American.

**Main Outcomes and Measures:**

The primary outcome was daily cumulative COVID-19 case rates. A secondary outcome was subsequent COVID-19 fatality rates, derived using mean cumulative fatality rates 21 to 28 days after each date. Multivariate regression models were estimated.

**Results:**

The final sample included 3023 pooled state- and day-level observations. The mean (SD) cumulative positive case rate was 103.186 (200.067) cases per 100 000 state population, the mean (SD) cumulative test rate was 744.23 (894.944) tests per 100 000 state population, and the mean (SD) subsequent cumulative fatality rate was 12.923 (21.737) deaths per 100 000 state population. There was a negative association of SAHOs with cumulative case rates (β = −1.166; 95% CI, −1.484 to −0.847; *P* < .001) and subsequent fatality rates (β = −0.204; 95% CI, −0.294 to −0.113; *P* < .001). Estimation analyses indicated that expected cumulative case rates would have been more than 200% higher and fatality rates approximately 22% higher if there were no SAHOs, as compared with SAHOs fully in place. A higher proportion of African American population was associated with higher case rates (β = 0.045; 95% CI, 0.014 to 0.077; *P* = .001) and fatality rates (β = 0.068; 95% CI, 0.044 to 0.091; *P* < .001).

**Conclusions and Relevance:**

In this cross-sectional study, SAHOs were associated with reductions in COVID-19 case rates. These findings could help inform policy makers to address the continued COVID-19 pandemic in the US. The proportion of African American population was positively associated with COVID-19 case rates, and this state-level finding adds to evidence from existing ecological studies using county-level data on racial disparities in COVID-19 infection rates and underlines the urgency of better understanding and addressing these disparities.

## Key Points

**Question:**

Are stay-at-home orders and state-level proportion of African American residents associated with coronavirus 2019 (COVID-19) infection rates?

**Findings:**

In this cross-sectional study including 3023 daily state-level observations from March to May 2020, results from multivariate regression models indicated that stay-at-home orders were associated with reductions in cumulative COVID-19 case rates. States with larger African American populations had higher COVID-19 case rates.

**Meaning:**

These findings underscore the importance of stay-at-home orders in addressing the COVID-19 pandemic and the need to address racial disparities in rates of infection.

## Teaser

This cross-sectional study examines the associations of state-level stay-at-home orders and proportion of African American population with coronavirus disease 2019 (COVID-19) case rates from March to May 2020.

## Introduction

The coronavirus disease (COVID-19) pandemic has shown little sign of abating in the US. As of August 17, 2020, there were 5 438 325 detected cases and 170 497 fatalities attributed to the disease.^1^ During March and April, most states in the US imposed shutdowns and enacted stay-at-home orders (SAHOs) in an effort to control the disease. However, mixed messages from political authorities on the policy usefulness, popular pressure, as well as concerns about the economic fallout^2^ led some states to lift SAHOs before public health experts considered it advisable. The subsequent increase in infections and fatalities in several states has led some experts to speculate that another round of shutdowns and SAHOs may be necessary to control the disease spread.^3^

Our understanding of the effectiveness of the initial SAHOs is still incomplete. One analysis in the *Wall Street Journal*^4^ suggested that SAHOs did not curb COVID-19 fatalities. Subsequent analysis, using small numbers of states, indicated that SAHOs might have helped curb infections and hospitalizations.^5,6^ We argue that a more comprehensive assessment of SAHOs that uses information from a wider selection of states will help inform public health experts and federal, state, and local policy makers.

Current understanding of racial disparities and COVID-19 is also incomplete. There is evidence that African American communities are disproportionately impacted by COVID-19,^7^ and analyses using zip code–level as well as county-level data have shown that areas with a high minority population, including higher African American population, have more COVID-19 cases and fatalities.^8,9,10,11^ Whether parallel associations between the African American population and COVID-19 outcomes also exist at the state level has not yet been explored, to our knowledge.

Our study has 2 aims. First, we used time-series, cross-sectional, daily, state-level data from March to May 2020, and leveraged the variations in the timing of SAHOs in different states as a natural experiment to assess the association between SAHOs and COVID-19 cases and subsequent fatalities. Second, we explored whether differences in the proportion of African American population between states was associated with state differences in COVID-19 cases and subsequent fatalities in our study period. Our findings contributed to the small but evolving body of literature on SAHOs, sociodemographic factors, and COVID-19 outcomes.

## Methods

### Data Source

This cross-sectional study used time series, cross-sectional, daily, state-level data from March to May 2020. The study used publicly available, aggregated, and deidentified data. The institutional review board of the University of Alabama at Birmingham approved the study as not human participant research.

Data on cumulative COVID-19 positive cases (hereafter, *cases*), cumulative COVID-19 tests (hereafter, *tests*), and cumulative fatalities for each day were obtained from the COVID Tracking Project .^12^ Initiated by *The Atlantic* in partnership with *Related Sciences*, the COVID Tracking Project collates data from state health agencies and makes it publicly available. *Cumulative* indicates the sum of all daily cases and tests reported for that state up to that day.

### Outcome Variables

Our main outcome variable was the cumulative case rate per 100 000 state population, measured daily. Our secondary outcome variable was the subsequent cumulative fatality rates. The emerging consensus among scientists is that COVID-19 fatalities manifest at least 2 weeks after onset of infection, and additional days are often required before deaths are verified as COVID-19 deaths.^13,14,15^ Hence, changes in fatalities corresponding to changes in cases will manifest in data a few weeks later. Therefore, we operationalized subsequent fatality rates as the mean cumulative fatality rate calculated over the 21st to 28th day after each date when cases were observed.

### Exposure Variables

The main exposure variable is a binary indicator for SAHOs.^16,17,18^ For each state, this was 1 for days when an SAHO was in effect, 0 for days it was not. Although states used different terms, such as *stay at home*, *safer at home*, *shelter-in-place*, and *healthy at home*, our study operationalized an SAHO as in effect when the state’s governor issued an order for residents of the entire state to leave home only for essential activities and when schools and nonessential businesses were closed. We hypothesized that SAHOs would be associated with a reduction in COVID-19 cases and as well as subsequent fatalities. The second exposure variable of interest is the proportion of African American population^19^ in the state. We also hypothesized that the proportion of African American population would be positively associated with COVID-19 cases and as well as subsequent fatalities.

### Other Control Variables

All regression models included cumulative test rates per 100 000 population. Given the variations in testing capacity and protocols for whom to test, both across states and within states over the study period,^20^ changes in detected cases following SAHOs may be an artifact of changes in testing; therefore, controlling for level of testing is critical. Models included a quadratic time-trend measured in days. Models included state-level characteristics that could potentially confound the association between outcomes and proportion of African American population—specifically, poverty rate,^21^ prevalence of obesity-related comorbidities,^22^ prevalence of asthma,^22^ and proportion of the population in urban areas.^23^ Finally, models controlled for other state-level characteristics that could potentially be associated with COVID-19 incidences, such as size of total state population,^24^ the share of population older than 65 years,^22^ certified nursing facilities^25,26^ per 100 000 population, and a binary indicator for mask mandates^27^ being instated.

### Statistical Analysis

Pooled state and day observations from March 1 to May 4, 2020, were used. States entered the sample on the first date they reported nonzero cumulative tests. The state of Washington, where the first test and case were reported on January 22, 2020, was excluded from the analysis. Data from all other states, as well as the District of Columbia (DC), were included. The included states and DC started first reporting nonzero cumulative tests between March 1 and March 12. Multivariate regression models were estimated with random intercepts to account for repeated daily observations from each state. Binary indicators for the first date each state started reporting tests and cases were also included (hereafter, *first date–fixed effects* (FDFE), as an earlier reporting date may indicate unmeasured characteristics, such as greater vigilance by state authorities in monitoring the disease.

Outcomes variables and cumulative test rates were log-transformed to minimize the influence of outliers, and any 0 values for cumulative cases and subsequent fatalities were replaced with (1/10 million) prior to log-transformation. Coefficient estimates for key results were interpreted as percentage changes in the outcome variable using the formula 100[*exp*(*β_j_*) − 1]. We considered 3 obesity-related comorbidities: the prevalence of obesity, diabetes, and hypertension. High multicollinearity between them (Variance Inflation Factor of ≥4) precluded including more than 1; hence, we selected diabetes as the control for obesity-related comorbidities. Equations for empirical models are shown in the eAppendix in the Supplement.

Two-sided *P* = .05 was set as the threshold for statistical significance for hypothesis testing. All models were estimated using Stata statistical software version 16 (StataCorp).

We conducted 5 supplementary sensitivity analyses. First, recognizing that COVID-19 cases are likely to be severe undercounts of true infection rates, we derived inferred infection rates using the subsequent fatality rates and 2 alternate estimates of infection-fatality ratio,^28,29^ as well as from daily detected cases combined with daily positivity ratios.^30^ We re-estimated our models alternatively using the 3 inferred infection rates in place of case rates. Second, we also re-estimated the models using daily incremental cases per 100 000 population. The motivation was to investigate whether findings on associations between SAHOs and daily cumulative case rates were consistent with findings on SAHOs and daily incremental case rates. Third, we tested if our main results changed when the state of Washington was included. Fourth, we tested whether our results changed if New York, the state with the highest number of cases in our study period, was excluded. Fifth, we explored whether the association between SAHOs and case rates changed over time by including interaction terms between SAHOs and time-trend in the model.^31^

## Results

**Figure 1.**
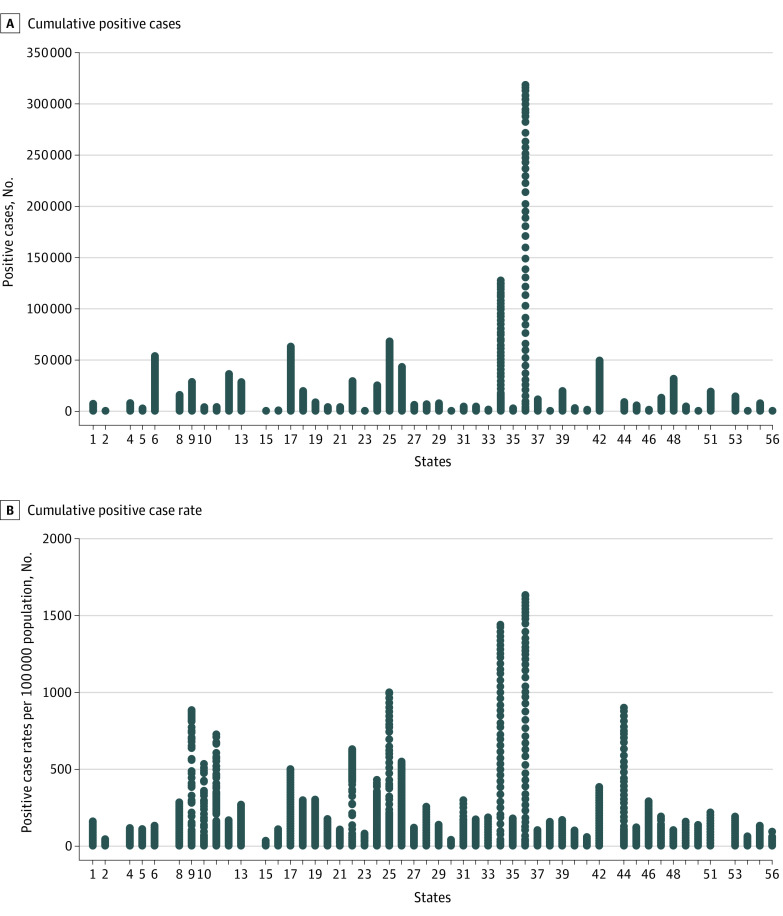
Cumulative Positive Case Counts and Case Rates by State States are indicated by Federal Information Processing Standard (FIPS) codes.

**Figure 2.**
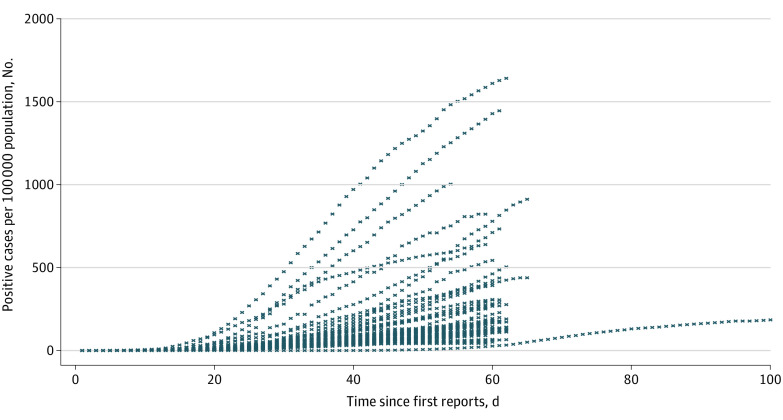
Trends in Cumulative Positive Cases Reported by the State For each state, day 1 is the first day when it reported a positive value for tests conducted. The state of Washington started reporting from January, and thus has a longer number of days than any other state and was omitted from the main analysis.

The final sample included 3023 state-day observations. Descriptive statistics show the mean (SD) cumulative case rate was 103.186 (200.067) cases per 100 000 population, and the mean (SD) cumulative test rate was 744.230 (894.944) tests per 100 000 population (Table 1). The corresponding mean (SD) cumulative fatality rate was 12.923 (21.737) deaths per 100 000 population. There were SAHOs in place for 1516 days (50.1%) in the overall sample. The mean (SD) proportion of African American populations in state populations was 11.081% (10.473%). Figure 1 presents cumulative case counts and case rates by state during the study period. The cumulative case rate as of May 4 ranged from 318 953 cases in New York to 370 cases in Alaska. New York and New Jersey are outliers in terms of case rates, justifying log-transformation of the outcome variable. Figure 2 presents trends in cumulative case rates for all states, including the state of Washington. A quadratic pattern is apparent in the trends.

**Table 1.** Descriptive Statistics

Variable	Mean (SD) [range], No. per 100 000 population
SAHO in place^a^	0.545 (0.498) [0-1]
African American, %	11.081 (10.473) [0-45]
Daily cumulative rate	
Positive cases	103.186 (200.067) [0-1639.561]
Tests	744.23 (894.944) [0-6998.181]
Fatalities	12.923 (21.737) [0-128.569]
Urban population	74.002 (14.777) [38.66-100]
Nursing facilities	5.647 (2.635) [1.98-13.851]
Total population	64.842 (74.277) [5.788-395.122]
Asthma, %	9.681 (1.226) [7.4-12.3]
Diabetes, %	11.146 (1.905) [7-16.2]
Age >65 y, %	16.477 (2.07) [11-21]
Poverty, %^b^	11.833 (2.817) [6.6-19.8]

Abbreviation: SAHO, stay-at-home order.

^a^ Classified as 1 if in place, 0 otherwise (2020).

^b^ Defined as individuals under poverty level per state.

eTable 1 in the Supplement presents a state-by-state breakdown of key model variables. Seven states did not impose SAHOs.^16^ Additionally, 12 states lifted their SAHOs before May 4. eFigure 1 in the Supplement presents the distributions across states of the proportion of days for which SAHOs were in place, including 0% of days for states that did not implement SAHOs, and eFigure 2 in the Supplement presents the distribution of the proportion of African American individuals in the state population.

Table 2 presents results from the random effects regression models for case rates with FDFE (model 1) and without FDFE (model 2) and subsequent fatality rates with FDFE (model 3) and without FDFE (model 4). Implementation of SAHOs was associated with a significant reduction in case rates (model 1: β = −1.166; 95% CI, −1.484 to −0.847; *P* < .001; model 2: β = −1.170; 95% CI, (−1.487 to −0.853; *P* < .001). Along parallel lines, SAHOs were associated with a significant reduction in fatality rates (model 3: β = −0.204; 95% CI, −0.294 to −0.113; *P* < .001; model 4: β = −0.204; 95% CI, −0.295 to −0.114; *P* < .001). After translating the coefficient estimates into percentage changes using 100[*exp*(*β_j_*) − 1], the results implied that having no SAHO, compared with a fully implemented SAHO, was associated with a mean of 218.9% (95% CI, 134.0%-339.3%) higher cumulative cases and 22.1% (95% CI, 12.1%-34.3%) higher cumulative fatalities over the study period.

**Table 2.** Multivariate Regression Analysis With State-Level Random Effects for Cumulative COVID-19 Case Rates and Subsequent Fatality Rates

Variable	Model 1: Cumulative with FDFE (n = 3023)		Model 2: Cumulative without FDFE (n = 3023)		Model 3: Cumulative fatalities with FDFE (n = 3012)^a^		Model 4: Cumulative fatalities without FDFE (n = 3012)^a^	
	β (95% CI)	*P* value	β (95% CI)	*P* value	β (95% CI)	*P* value	β (95% CI)	*P* value
SAHO in place	−1.166 (−1.484 to −0.847)	<.001	−1.170 (−1.487 to −0.853)	<.001	−0.204 (−0.294 to −0.113)	<.001	−0.204 (−0.295 to −0.114)	<.001
African American, proportion of population	0.045 (0.014 to 0.077)	.005	0.048 (0.019 to 0.077)	.001	0.068 (0.044 to 0.091)	<.001	0.064 (0.041 to 0.088)	<.001
Log cumulative test rates	0.615 (0.557 to 0.673)	<.001	0.615 (0.557 to 0.673)	<.001	0.165 (0.149 to 0.181)	<.001	0.165 (0.149 to 0.182)	<.001
Mask order	−0.026 (−0.627 to 0.575)	.93	−0.045 (−0.643 to 0.554)	.88	−0.059 (−0.229 to 0.111)	.50	−0.063 (−0.233 to 0.106)	.46
Nursing facilities per 100 000 population	0.043 (−0.078 to 0.165)	.49	0.061 (−0.051 to 0.172)	.29	0.092 (0.003 to 0.181)	.04	0.133 (0.043 to 0.224)	.004
Total population	0.005 (0 to 0.010)	.04	0.006 (0.002 to 0.009)	.005	0.004 (0.001 to 0.008)	.02	0.003 (0 to 0.006)	.05
Urban population, % of total population	0.037 (0.012 to 0.062)	.004	0.042 (0.020 to 0.064)	<.001	0.032 (0.014 to 0.050)	<.001	0.040 (0.022 to 0.058)	<.001
Days	0.433 (0.395 to 0.472)	<.001	0.434 (0.395 to 0.472)	<.001	0.077 (0.066 to 0.087)	<.001	0.077 (0.066 to 0.087)	<.001
Days-squared	−0.005 (−0.006 to −0.005)	<.001	−0.005 (−0.006 to −0.005)	<.001	−0.001 (−0.001 to −0.001)	<.001	−0.001 (−0.001 to −0.001)	<.001
Asthma, % of total population	0.197 (−0.070 to 0.464)	.15	0.253 (0.018 to 0.487)	.03	0.280 (0.085 to 0.475)	.005	0.363 (0.174 to 0.552)	<.001
Diabetes, % of total population	0.025 (−0.185 to 0.235)	.82	−0.026 (−0.222 to 0.171)	.80	−0.066 (−0.220 to 0.088)	.40	−0.061 (−0.220 to 0.098)	.46
Age >65 y, % of total population	0.030 (−0.122 to 0.183)	.70	0.050 (−0.096 to 0.196)	.50	0.079 (−0.032 to 0.191)	.16	0.071 (−0.047 to 0.189)	.24
Poverty, % of total population	−0.134 (−0.263 to −0.006)	.04	−0.117 (−0.239 to 0.004)	.06	−0.037 (−0.131 to 0.057)	.44	−0.033 (−0.131 to 0.065)	.51
*R*^2^								
Within	0.71	NA	0.71	NA	0.68	NA	0.68	NA
Between	0.58	NA	0.53	NA	0.54	NA	0.48	NA
ρ	0.08	NA	0.08	NA	0.42	NA	0.46	NA

Abbreviations: FDFE, first date fixed effects; NA, not applicable; SAHO, stay-at-home order.

^a^ For each day in the sample, the subsequent cumulative fatality rates were operationalized using mean cumulative fatality rates 21 to 28 days after that date. Outcome variables were log-transformed, with values of 0 replaced with (1/10 million) prior to log transformation.

States with higher proportions of African American populations were associated with higher case rates (model 1: β =  0.045; 95% CI, 0.014 to 0.077; *P* = .005; model 2: β = 0.048; 95% CI, 0.019 to 0.077; *P* = .001) and fatality rates (model 3: β = 0.068; 95% CI, 0.044 to 0.091; *P* < .001; model 4: β = 0.064; 95% CI, 0.041 to 0.088; *P* < .001). Converted to percentage changes, this implied that a 1–percentage point increase in a state’s African American population was associated with a mean of 4.6% (95% CI, 1.4%-8.0%) higher cumulative cases and 7.0% (95% CI, 4.5%-9.5%) higher fatalities.

With respect to the control variables, there was a quadratic trend in both cumulative case rates and fatality rates, with the coefficient estimate of days being positive and significant and days-squared being negative and significant (Table 2). The natural log of cumulative test rates and share of the urban population was significantly associated with increased case rates and subsequent fatality rates. The prevalence of asthma and the number of nursing facilities per 100 000 population were significantly associated with increased fatality rates. (Table 2)

Expected estimated values of cumulative positive case rates with no SAHOs (SAHO = 0) vs with SAHOs (SAHO = 1) at different intervals in time were computed. Estimated cumulative case rates were 2.5- to 3-fold higher in the absence of SAHOs than when SAHOs were present, holding all other model covariates equal (Table 3).

**Table 3.** Estimated Cumulative Case Rates in Hypothetical Scenarios of No SAHOs and Full SAHOs

Days since first reporting	Expected estimated value of cumulative case rate^a^	
	Without SAHOs	With SAHOs
1	0.083	0.025
5	0.418	0.130
10	2.497	0.775
15	11.572	3.592
20	41.618	12.920
25	116.144	36.057
30	251.511	78.081
35	422.632	131.206
40	551.078	171.082
45	557.582	173.101

Abbreviation: SAHO, stay-at-home order.

^a^ Log values were retransformed to levels using exponentiation to obtain estimated values.

Results from supplementary analysis were in concordance with these findings. Models using the natural log of cumulative inferred infection rates as outcomes found SAHOs were significantly associated with reductions in the inferred infection rates, and a higher proportion of African American populations were associated with higher inferred infection rates. Also, the cumulative inferred infections (eTable 2 and eFigure 3 in the Supplement) were between 9.75- to 19.6-fold higher than reported cumulative cases. Further, in models using the natural log of daily incremental case rates, SAHOs were associated with a reduction in these case rates (eTable 3 in the Supplement). Inclusion of Washington state or exclusion of New York state did not significantly change the key results pertaining to the exposure variables (eTable 4 in the Supplement). Finally, the negative association between SAHOs and case rates and fatality rates was strengthened over time, as evidenced by the negative and significant coefficient estimate of the interaction of SAHOs and the time-trend (eTable 5 in the Supplement).

## Discussion

During March and April 2020, most US states imposed SAHOs in an effort to curb the spread of COVID-19. Substantial increases in COVID-19 cases and deaths in several parts of the US in recent months have led some public health experts to speculate whether another round of SAHOs may be required to fight the disease. However, the body of empirical studies on the effectiveness of the initial SAHOs is small. Our cross-sectional study added to this literature by conducting a comprehensive analysis with SAHOs as the main exposure variable that used all states (except Washington) and DC from March 1 to May 4, 2020. We used variations in the timings of imposition and lifting of SAHOs across states and the fact that some states did not impose an SAHO at all as a natural experiment to estimate the association of SAHOs with cumulative COVID-19 case rates and with subsequent fatality rates. We found that SAHOs were associated with reduced cumulative case rates and subsequent fatality rates. Our results indicated that a scenario of no SAHOs over this time period would have had 220% higher cumulative case rates and 22.1% percent higher cumulative fatality rates compared with a scenario of the full imposition of SAHOs. These findings were supported by supplementary analyses that showed that SAHOs were associated with a reduction in daily incremental case rates. Furthermore, recognizing the scientific consensus that detected case rates may substantially underestimate actual infection rates, we derived inferred infection rates using alternate estimates of the infection fatality rates,^28,29^ and using daily detected cases in conjunction with test positivity rates.^30^ We continued to find negative associations with SAHOs when we substituted the inferred infection rates as the outcome variable.

The observational nature of our study precludes causal inferences. However, the multivariate analyses that controlled for several other state-level characteristics and the consistent findings across cumulative case rates, cumulative fatality rates, daily incremental case rates, and inferred infection rates suggest that SAHOs were associated curbing disease spread. Our findings are also congruent with studies that have assessed the effectiveness of SAHOs using Illinois (which imposed an SAHO) vs Iowa (which did not),^5^ using hospitalizations trends in Colorado, Minnesota, Ohio, and Virginia,^6^ and using bivariate pre-SAHO vs post-SAHO comparison in 42 states for March 19 to April 7.^32^

Our second exposure variable of interest was the proportion of the African American population in the state. We found statistically significant and positive associations with this variable and COVID-19 case rates and subsequent fatality rates. The magnitude of the association was larger for fatality rates than case rates. These findings complemented existing county-level analyses of proportion of African American populations and COVID-19 outcomes.^9,10,11^ Furthermore, these associations were found in models that controlled for potential confounders, such as state-level poverty, prevalence of asthma, prevalence of diabetes (which was highly correlated with the prevalence of obesity and hypertension), and percentage of the state population in urban areas. It is well established that African American individuals disproportionately experience poverty and higher burdens of chronic disease and are more likely to live in urban areas than their White counterparts. However, our results indicated that these factors could not fully explain the association between African American race and adverse COVID-19 outcomes.

In face of this strengthening evidence of racial disparities in COVID-19 outcomes, future studies should explore the role of other measurable factors, such as being uninsured, housing and working conditions, or disproportionate representation in prisons and detention centers, as drivers of these disparities. Furthermore, we add our voices to those urging the scientific community to consider the role of structural and institutionalized racism as a potential driver for residual COVID-19 health disparities between racial groups,^33^ including potential lack of culturally competent health care, conscious or unconscious biases on the part of clinicians, and prior negative experiences with health care that lead to delay in care-seeking.^34^ We caution against premature inferences about a biologic basis of racial disparities or place-based stigma than can perpetuate harmful myths and misunderstandings and undermine the goal of eliminating health inequities.^35^

Some of our control variables yielded unintuitive results. For example, state poverty rates had a counterintuitive negative association with case rates in one model, although it was statistically insignificant in the others; and diabetes prevalence had no statistical significance. This could be an artifact of limited between-state variation in these variables, and more granular data may yield different results. However, a county-level study by Finch and Hernández Finch^20^ also reported similar counterintuitive results: in the month of April, it was more affluent counties reporting more COVID-19 cases. Another county-level study by Millett et al^11^ has reported negative associations between unemployment rates. which are typically correlated with poverty rates, and COVID-19 cases. This latter study also found no significant associations between diabetes prevalence, death rates from heart disease and hypertension, and COVID-19 case and death rates at the county level. Thus, further research with different data sets is called for to explore what ecological risk factors are associated with COVID-19 outcomes.^29^

### Limitations

Our study has several limitations. First, how strongly SAHOs were enforced and adhered to and what types of businesses were deemed essential and allowed to stay open may have varied across states. Second, we could not control for local stay-at-home ordinances at the city or county level, owing to a lack of reliable information on such ordinances or what proportion of the state’s population was impacted by them. Third, testing capacity, protocols, and the speed and accuracy of reporting tests, positive cases, and fatalities may not have been uniform across states or over time. Fourth, we operationalize subsequent fatality rates as those occurring 21 to 28 days after cases, and results might be sensitive to alternate specifications. Fifth, our supplemental findings using inferred infection rates should be treated with caution, as the science on how to estimate true underlying infections from existing information is still evolving; additionally, infection-fatality ratios can vary by age and race, but breakdowns by age and race for cases, tests, or fatalities were not available during this study period. Sixth, since our focus was on SAHOs, we did not consider patterns in COVID-19 cases and fatalities from more recent months. We intend to explore that, as well as COVID-19 hospitalization patterns, in future research.

## Conclusions

The COVID-19 situation remains precarious in the US, even as several schools and universities start reopening across the country in in-person mode. Other places in the world, such as Melbourne, Australia, and Auckland, New Zealand, have imposed second rounds of SAHOs owing to a resurgence of infections. While the high economic cost makes SAHOs unsustainable as long-term policy, our findings could help inform federal, state, and local policy makers in weighing the costs and benefits of different short-term options to combat the pandemic. Our findings also emphasize the importance of understanding and addressing the drivers of racial disparities in COVID-19 outcomes as part of the overarching goal of improving health equity in the US.

## Notes

Supplement. **eTable 1.** State-by-State Breakdown of All Variables **eTable 2.** Models With Cumulative Inferred Infection Rates as Outcomes **eTable 3.** Models with Daily Incremental Case Rates as Outcomes **eTable 4.** Sensitivity Analysis With the Main Model **eTable 5.** Models that Include the Interaction of SAHOs With Time Trend **eFigure 1.** Distribution of Percentage of Days Each State Imposed State-Level Stay at Home Orders **eFigure 2.** Distribution of Percentage of African American Population in Each State **eFigure 3.** Comparison of Reported COVID-19 Cases and Inferred Infections **eAppendix.** Multivariate Regression Model With State-Specific Random Effects **eReferences.**
