# Supplementary material for: Association of State Stay-at-Home Orders and State-Level African American Population With COVID-19 Case Rates
Source: JAMA Netw Open. 2020 Oct 23;3(10):e2026010. doi: 10.1001/jamanetworkopen.2020.26010 (PMC7584926; doi:10.1001/jamanetworkopen.2020.26010)
Supplement: Supplement. — eTable 1. State-by-State Breakdown of All Variables eTable 2. Models With Cumulative Inferred Infection Rates as Outcomes eTable 3. Models with Daily Incremental Case Rates as Outcomes eTable 4. Sensitivity Analysis With the Main Model eTable 5. Models that Include the Interaction of SAHOs With Time Trend eFigure 1. Distribution of Percentage of Days Each State Imposed State-Level Stay at Home Orders eFigure 2. Distribution of Percentage of African American Population in Each State eFigure 3. Comparison of Reported COVID-19 Cases and Inferred Infections eAppendix. Multivariate Regression Model With State-Specific Random Effects eReferences. [file jamanetwopen-e2026010-s001.pdf]

Supplemental Online Content

Padalabalanarayanan S, Hanumanthu VS, Sen B. Association of state stay-at-home orders and state-level African American population with COVID-19 case rates. *JAMA Netw Open*. 2020;3(10):e2026010. doi:10.1001/jamanetworkopen.2020.26010

eTable 1. State-by-State Breakdown of All Variables

eTable 2. Models With Cumulative Inferred Infection Rates as Outcomes

eTable 3. Models with Daily Incremental Case Rates as Outcomes

eTable 4. Sensitivity Analysis With the Main Model

eTable 5. Models that Include the Interaction of SAHOs With Time Trend

eFigure 1. Distribution of Percentage of Days Each State Imposed State-Level Stay at Home Orders

eFigure 2. Distribution of Percentage of African American Population in Each State

eFigure 3. Comparison of Reported COVID-19 Cases and Inferred Infections

eAppendix. Multivariate Regression Model With State-Specific Random Effects

eReferences.

This supplemental material has been provided by the authors to give readers additional information about their work.

© 2020 Padalabalanarayanan S et al. *JAMA Network Open.*

eTable 1. State-by-State Breakdown of All Variables

Table 1. State-by-state breakdown of all variables

State	SAHO Imposed date	African American	Mean positive case rate	Mean total test rate	First Date of reporting	SAHO lifted(1)	Total Population	Urban Population	Mask order Date	Nursing homes	Asthma	Diabetes	Above-65	Poverty
Alabama	4/4/2020	25.30	163.67	2103.06	3/7/2020	4/30/2020	4903185	59.04	No orders	228	10.50	14.50	17	15.60
Alaska	3/28/2020	4.20	50.58	2969.47	3/6/2020	4/24/2020	731545	66.02	No orders	18	9.20	8.80	12	12.60
Arizona	3/31/2020	4.10	122.54	1171.26	3/4/2020	N/A	7278717	89.81	No orders	145	10.00	10.80	18	13.20
Arkansas	No orders	14.30	114.59	1804.03	3/6/2020	N/A	3017804	56.16	No orders	231	9.80	13.90	17	15.40
California	3/19/2020	5.60	139.04	1892.77	3/4/2020	N/A	39512223	94.95	No orders	1198	8.50	10.40	14	12.00
Colorado	3/26/2020	3.20	288.87	1442.85	3/5/2020	4/27/2020	5758736	86.15	No orders	221	9.10	7.00	14	9.00
Connecticut	3/23/2020	9.70	821.45	2874.75	3/7/2020	N/A	3565287	87.99	4/20/2020	223	10.30	9.70	17	10.40
Delaware	3/24/2020	20.40	543.05	2475.96	3/6/2020	N/A	973764	83.3	4/28/2020	45	10.10	11.90	19	8.10
District of Columbia	4/1/2020	41.70	732.56	3371.60	3/5/2020	N/A	705749	100	No orders	18	11.60	8.40	12	14.30
Florida	4/3/2020	14.10	171.79	2068.51	3/4/2020	N/A	21477737	91.16	No orders	690	8.70	12.60	21	13.50
Georgia	4/3/2020	30.10	276.60	1726.64	3/4/2020	4/25/2020	10617423	75.07	No orders	359	8.90	12.60	14	14.00
Hawaii	3/25/2020	1.70	43.79	2391.88	3/7/2020	N/A	1415872	91.93	4/20/2020	42	9.30	11.50	19	9.90
Idaho	3/25/2020	0.80	115.33	1667.93	3/7/2020	5/1/2020	1787065	70.58	No orders	71	8.70	10.20	16	11.50
Illinois	3/21/2020	13.40	503.79	2629.04	3/4/2020	N/A	12671821	88.49	5/1/2020	731	8.70	10.00	15	10.90
Indiana	3/24/2020	8.70	304.61	1682.91	3/6/2020	N/A	6732219	72.44	No orders	552	10.00	12.50	16	11.60
Iowa	No orders	3.20	307.54	1811.72	3/6/2020	No orders	3155070	64.02	No orders	437	7.90	10.00	17	8.20
Kansas	3/30/2020	5.50	180.04	1325.05	3/6/2020	N/A	2913314	74.2	No orders	276	9.80	11.60	16	10.90
Kentucky	3/26/2020	7.70	114.82	1307.37	3/6/2020	N/A	4467673	58.38	No orders	285	11.50	13.70	16	14.60
Louisiana	3/23/2020	30.20	638.29	3892.00	3/7/2020	N/A	4648794	73.19	No orders	277	8.90	14.10	15	19.80
Maine	4/2/2020	1.40	89.64	1543.73	3/7/2020	5/1/2020	1344212	38.66	5/1/2020	100	12.30	10.60	21	12.00
Maryland	3/30/2020	28.80	436.81	2266.00	3/5/2020	N/A	6045680	87.2	20200418	226	9.30	12.00	15	7.80
Massachusetts	3/24/2020	6.40	1002.35	4704.65	3/12/2020	N/A	6892503	91.97	No orders	399	10.20	8.60	17	10.00
Michigan	3/24/2020	13.00	438.12	2224.85	3/1/2020	N/A	9986857	74.57	No orders	443	11.20	11.70	17	11.00
Minnesota	3/27/2020	5.40	128.27	1523.88	3/6/2020	N/A	5639632	73.27	No orders	375	8.30	8.90	16	8.20
Mississippi	4/3/2020	35.40	264.67	2677.18	3/7/2020	4/27/2020	2976149	49.35	No orders	204	9.70	14.40	16	19.00
Missouri	4/6/2020	10.90	142.63	1490.05	3/7/2020	N/A	6137428	70.44	No orders	518	9.40	11.50	17	11.90
Montana	3/28/2020	1.00	42.76	1411.71	3/7/2020	4/26/2020	1068778	55.89	No orders	72	10.00	9.40	19	10.30
Nebraska	No orders	4.30	305.52	1744.93	3/5/2020	No orders	1934408	73.13	No orders	214	8.90	9.70	16	11.00
Nevada	4/1/2020	8.80	178.27	1498.82	3/5/2020	N/A	3080156	94.2	No orders	61	8.00	10.80	16	13.10
New Hampshire	3/27/2020	1.20	190.33	1976.16	3/4/2020	N/A	1359711	60.3	No orders	74	11.80	10.30	18	6.60
New Jersey	3/21/2020	12.50	1444.11	3121.08	3/5/2020	N/A	8882190	94.68	4/8/2020	364	8.40	10.80	16	9.10
New Mexico	3/24/2020	1.60	183.61	3718.81	3/6/2020	N/A	2096829	77.43	No orders	74	9.90	12.50	18	18.20
New York	3/22/2020	14.00	1639.56	5178.02	3/4/2020	N/A	19453561	87.87	4/17/2020	609	10.10	11.00	16	11.90
North Carolina	3/30/2020	20.60	112.97	1396.24	3/4/2020	N/A	10488084	66.09	No orders	429	9.40	12.50	16	14.10
North Dakota	No orders	3.50	160.75	4560.52	3/7/2020	No orders	762062	59.9	No orders	80	8.20	9.40	15	11.10
Ohio	3/23/2020	11.70	175.15	1327.35	3/5/2020	N/A	11689100	77.92	No orders	966	9.50	12.20	17	12.40
Oklahoma	3/24/2020	7.10	102.20	1613.56	3/7/2020	4/24/2020	3956971	66.24	No orders	303	10.30	12.50	16	13.10
Oregon	3/23/2020	1.80	65.41	1504.53	3/4/2020	N/A	4217737	81.03	No orders	136	11.60	11.00	18	10.60
Pennsylvania	4/1/2020	10.10	391.28	1918.37	3/6/2020	N/A	12801989	78.66	4/19/2020	693	10.00	11.30	18	11.40
Rhode Island	3/28/2020	5.00	911.12	6998.18	3/1/2020	N/A	1059361	90.73	No orders	83	11.90	10.90	17	10.10
South Carolina	4/7/2020	25.20	128.69	1246.68	3/4/2020	4/20/2020	5148714	66.33	No orders	191	9.10	13.30	18	14.00
South Dakota	No orders	1.40	301.59	2115.28	3/7/2020	No orders	884659	56.65	No orders	108	7.90	9.30	16	10.70
Tennessee	4/2/2020	15.70	198.72	3096.17	3/5/2020	4/27/2020	6829174	66.39	No orders	314	9.80	13.80	16	11.70
Texas	4/2/2020	11.80	111.51	1405.02	3/4/2020	5/1/2020	28995881	84.7	No orders	1227	7.40	12.60	13	13.40
Utah	No orders	1.00	165.85	3888.42	3/7/2020	No orders	3205958	90.58	No orders	99	9.30	8.40	11	7.80
Vermont	3/25/2020	0.80	144.55	2777.61	3/6/2020	N/A	623989	38.9	No orders	36	12.00	9.20	20	9.20
Virginia	3/30/2020	18.40	228.36	1315.60	3/5/2020	N/A	8535519	75.45	No orders	286	8.50	10.50	16	10.10
Washington	3/23/2020	3.20	199.41	2784.08	1/22/2020	N/A	7614893	84.05	No orders	217	9.60	9.90	16	9.70
West Virginia	3/24/2020	3.20	67.29	2970.68	3/6/2020	N/A	1792147	48.72	No orders	123	12.30	16.20	20	16.50
Wisconsin	3/25/2020	5.60	141.45	1523.47	3/4/2020	N/A	5822434	70.15	No orders	374	9.00	8.70	17	8.90
Wyoming	No orders	0.00	101.25	1806.28	3/7/2020	No orders	578759	64.76	No orders	38	8.70	8.80	17	11.20

SAHO Lifted[1]

© 2020 Padalabalanarayanan S et al. *JAMA Network Open.*

eTable 2. Models With Cumulative Inferred Infection Rates as Outcomes

**Inferred Infection Rate-1[a]**								
	With FDFE (N=3012)				Without FDFE (N=3012)			
Variable	*β*	*p*	*95% Confidence interval*		*β*	*p*	*95% Confidence interval*	
SAHO in place	-0.258	0.000*	-0.372	-0.143	-0.257	0.000*	-0.371	-0.142
African American	0.069	0.000*	0.043	0.094	0.066	0.000*	0.041	0.091
Log cumulative tests	0.211	0.000*	0.191	0.232	0.212	0.000*	0.191	0.232
Mask order	-0.076	0.489	-0.292	0.140	-0.080	0.466	-0.295	0.135
Nursing Facilities	0.096	0.054	-0.001	0.194	0.136	0.005*	0.040	0.231
Total Population	0.004	0.022*	0.001	0.008	0.003	0.049*	0.000	0.007
Urban population	0.033	0.001*	0.013	0.053	0.040	0.000*	0.021	0.059
days	0.064	0.000*	0.051	0.078	0.064	0.000*	0.050	0.078
$(days)^2$	-0.001	0.000*	-0.001	0.000	-0.001	0.000*	-0.001	0.000
Asthma	0.295	0.007*	0.081	0.509	0.376	0.000*	0.176	0.576
Diabetes	-0.043	0.619	-0.211	0.126	-0.037	0.669	-0.204	0.131
Above-65	0.073	0.239	-0.049	0.196	0.065	0.305	-0.059	0.190
Poverty	-0.044	0.399	-0.148	0.059	-0.042	0.429	-0.145	0.062
R-squared within	0.594				0.594			
R-squared between	0.525				0.480			
rho	0.346				0.368			

© 2020 Padalabalanarayanan S et al. *JAMA Network Open.*

Inferred Infection Rate-2[b]								
	With FDFE (N=3012)				Without FDFE (N=3012)			
Variable	*β*	*p*	*95% Confidence interval*		*β*	*p*	*95% Confidence interval*	
SAHO in place	-0.266	0.000*	-0.385	-0.147	-0.265	0.000*	-0.383	-0.146
African American	0.069	0.000*	0.043	0.095	0.066	0.000*	0.041	0.091
Log cumulative tests	0.219	0.000*	0.197	0.240	0.219	0.000*	0.198	0.240
Mask order	-0.079	0.488	-0.302	0.144	-0.083	0.467	-0.306	0.140
Nursing Facilities	0.097	0.056	-0.002	0.196	0.136	0.006*	0.040	0.233
Total Population	0.004	0.023*	0.001	0.008	0.003	0.048*	0.000	0.007
Urban population	0.033	0.001*	0.013	0.053	0.040	0.000*	0.021	0.060
days	0.063	0.000*	0.048	0.077	0.062	0.000*	0.048	0.077
$(days)^2$	-0.001	0.000*	-0.001	0.000	-0.001	0.000*	-0.001	0.000
Asthma	0.298	0.007*	0.081	0.515	0.378	0.000*	0.176	0.580
Diabetes	-0.039	0.655	-0.210	0.132	-0.033	0.705	-0.202	0.137
Above-65	0.073	0.252	-0.052	0.197	0.064	0.317	-0.061	0.190
Poverty	-0.046	0.393	-0.150	0.059	-0.043	0.418	-0.148	0.061
R-squared within	0.581				0.581			
R-squared between	0.523				0.478			
rho	0.337				0.356			

© 2020 Padalabalanarayanan S et al. *JAMA Network Open.*

Inferred Infection Rate-3[c]								
	With FDFE (N=3023)				Without FDFE (N=3023)			
Variable	*β*	*p*	*95% Confidence interval*		*β*	*p*	*95% Confidence interval*	
SAHO in place	-1.500	0.000*	-1.818	-1.182	-1.483	0.000*	-1.800	-1.166
African American	0.070	0.000*	0.041	0.098	0.067	0.000*	0.041	0.093
Log cumulative tests	0.517	0.000*	0.459	0.575	0.519	0.000*	0.461	0.577
Mask order	0.243	0.429	-0.359	0.845	0.269	0.378	-0.330	0.868
Nursing Facilities	0.066	0.241	-0.044	0.176	0.116	0.025*	0.015	0.216
Total Population	0.008	0.000*	0.004	0.012	0.006	0.001*	0.002	0.009
Urban population	0.034	0.003*	0.012	0.057	0.040	0.000*	0.020	0.060
days	0.585	0.000*	0.547	0.624	0.584	0.000*	0.545	0.622
$(days)^2$	-0.007	0.000*	-0.007	-0.006	-0.007	0.000*	-0.007	-0.006
Asthma	0.250	0.043*	0.008	0.492	0.307	0.004*	0.095	0.518
Diabetes	-0.077	0.431	-0.267	0.114	-0.101	0.265	-0.278	0.076
Above-65	0.101	0.153	-0.037	0.239	0.094	0.162	-0.038	0.225
Poverty	-0.095	0.110	-0.212	0.022	-0.068	0.221	-0.178	0.041
R-squared within	0.742				0.724			
R-squared between	0.589				0.518			
rho	0.065				0.061			

[a]Inferred infection assuming infection fatality rate of 1.3%[2]. [b]Inferred infection assuming infection fatality rate of 0.65%[3]. [c]Derived using daily positive cases and daily positive ratio[4]

© 2020 Padalabalanarayanan S et al. *JAMA Network Open.*

eTable 3. Models with Daily Incremental Case Rates as Outcomes

Multivariate Regression Analysis with State Random Effects, for Daily Incremental COVID-19 Cases								
**Washington State Included**								
	With FDFE (N=2993)				Without FDFE (N=2993)			
Variable	*β*	*p*	*95% Confidence interval*		*β*	*p*	*95% Confidence interval*	
SAHO in place	-0.233	0.148	-0.549	0.083	-0.216	0.179	-0.530	0.099
African American	0.050	0.000*	0.025	0.074	0.050	0.000*	0.027	0.072
Log cumulative tests	0.380	0.000*	0.365	0.394	0.381	0.000*	0.366	0.395
Mask order	-0.201	0.514	-0.803	0.401	-0.201	0.510	-0.800	0.398
Nursing Facilities	0.104	0.028*	0.011	0.197	0.144	0.001*	0.056	0.232
Total Population	0.002	0.389	-0.002	0.005	0.000	0.888	-0.003	0.003
Urban population	0.026	0.008*	0.007	0.045	0.033	0.000*	0.016	0.050
Days	0.378	0.000*	0.347	0.408	0.376	0.000*	0.346	0.407
$(\text{days})^2$	-0.005	0.000*	-0.005	-0.004	-0.005	0.000*	-0.005	-0.004
Asthma	0.040	0.702	-0.164	0.244	0.097	0.303	-0.087	0.280
Diabetes	-0.044	0.589	-0.204	0.116	-0.069	0.378	-0.223	0.085
Above-65	0.029	0.620	-0.087	0.146	0.027	0.640	-0.087	0.141
Poverty	-0.042	0.402	-0.141	0.056	-0.027	0.580	-0.122	0.068
R-squared within	0.762				0.726			
R-squared between	0.806				0.771			
rho	0.042				0.043			

© 2020 Padalabalanarayanan S et al. *JAMA Network Open.*

eTable 4. Sensitivity Analysis With the Main Model

**a. Multivariate Regression Analysis with State Random Effects, for Cumulative COVID-19 Cases and Subsequent Fatalities**																
**New York Excluded**																
	Cumulative with FDFE (N=2961)				Cumulative without FDFE (N=2961)				Cumulative fatalities with FDFE (N=2950)				Cumulative fatalities without FDFE (N=2950)			
Variable	*β*	*p*	*95% Confidence interval*		*β*	*p*	*95% Confidence interval*		*β*	*p*	*95% Confidence interval*		*β*	*p*	*95% Confidence interval*	
SAHO in place	-1.166	0.000 *	-1.489	-0.844	-1.168	0.000 *	-1.490	-0.847	-0.200	0.000 *	-0.292	-0.108	-0.201	0.000 *	-0.293	-0.109
African American	0.045	0.006 *	0.013	0.077	0.047	0.001 *	0.018	0.076	0.066	0.000 *	0.043	0.090	0.064	0.000 *	0.040	0.087
Log cumulative tests	0.613	0.000 *	0.554	0.671	0.613	0.000 *	0.555	0.671	0.165	0.000 *	0.148	0.181	0.165	0.000 *	0.148	0.181
Mask order	-0.024	0.942	-0.676	0.628	-0.043	0.896	-0.692	0.606	-0.014	0.880	-0.198	0.170	-0.017	0.857	-0.201	0.167
Nursing Facilities	0.040	0.521	-0.083	0.163	0.061	0.284	-0.051	0.173	0.087	0.057	-0.003	0.176	0.135	0.004 *	0.043	0.226
Total Population	0.004	0.070	0.000	0.009	0.005	0.017 *	0.001	0.009	0.003	0.067	0.000	0.007	0.002	0.209	-0.001	0.005
Urban population	0.036	0.005 *	0.011	0.061	0.042	0.000 *	0.020	0.064	0.030	0.001 *	0.012	0.048	0.039	0.000 *	0.021	0.057
days	0.438	0.000 *	0.399	0.477	0.438	0.000 *	0.399	0.477	0.077	0.000 *	0.066	0.088	0.077	0.000 *	0.066	0.088
$(\text{days})^2$	-0.005	0.000 *	-0.006	-0.005	-0.005	0.000 *	-0.006	-0.005	-0.001	0.000 *	-0.001	-0.001	-0.001	0.000 *	-0.001	-0.001
Asthma	0.166	0.233	-0.107	0.439	0.232	0.055	-0.005	0.468	0.225	0.027 *	0.026	0.423	0.329	0.001 *	0.136	0.521
Diabetes	0.033	0.761	-0.179	0.245	-0.017	0.863	-0.214	0.180	-0.051	0.514	-0.206	0.103	-0.047	0.563	-0.208	0.113
Above-65	0.032	0.686	-0.122	0.185	0.050	0.505	-0.096	0.195	0.081	0.155	-0.031	0.193	0.070	0.249	-0.049	0.188
Poverty	-0.135	0.041 *	-0.265	-0.005	-0.119	0.055	-0.240	0.002	-0.038	0.432	-0.132	0.057	-0.035	0.490	-0.134	0.064
R-squared within	0.708				0.708				0.682				0.682			

© 2020 Padalabalanarayanan S et al. *JAMA Network Open.*

R-squared between	0.548				0.489				0.547				0.475			
rho	0.081				0.077				0.414				0.459			

© 2020 Padalabalanarayanan S et al. *JAMA Network Open.*

b. Multivariate Regression Analysis with State Random Effects, for Cumulative COVID-19 Cases and Subsequent Fatalities																
**Washington State Included**																
	Cumulative with FDFE (N=3088)				Cumulative without FDFE (N=3088)				Cumulative fatalities with FDFE (N=3077)				Cumulative fatalities without FDFE (N=3077)			
Variable	*β*	*p*	*95% Confidence interval*		*β*	*p*	*95% Confidence interval*		*β*	*p*	*95% Confidence interval*		*β*	*p*	*95% Confidence interval*	
SAHO in place	-0.444	0.004 *	-0.748	-0.140	-0.461	0.003 *	-0.765	-0.158	-0.120	0.005 *	-0.205	-0.036	-0.123	0.004 *	-0.207	-0.039
African American	0.047	0.004 *	0.015	0.079	0.048	0.002 *	0.017	0.079	0.068	0.000 *	0.045	0.091	0.064	0.000 *	0.040	0.089
Log cumulative tests	0.765	0.000 *	0.711	0.819	0.764	0.000 *	0.710	0.819	0.182	0.000 *	0.167	0.197	0.182	0.000 *	0.167	0.197
Mask order	-0.645	0.037 *	-1.250	-0.040	-0.706	0.022 *	-1.308	-0.104	-0.130	0.128	-0.297	0.037	-0.137	0.107	-0.303	0.029
Nursing Facilities	0.064	0.301	-0.058	0.186	0.065	0.283	-0.054	0.183	0.094	0.038 *	0.005	0.184	0.134	0.006 *	0.039	0.229
Total Population	0.005	0.058	0.000	0.009	0.005	0.019 *	0.001	0.009	0.004	0.018 *	0.001	0.008	0.003	0.076	0.000	0.006
Urban population	0.034	0.006 *	0.010	0.059	0.039	0.001 *	0.015	0.062	0.032	0.001 *	0.014	0.050	0.039	0.000 *	0.020	0.058
days	0.252	0.000 *	0.224	0.281	0.251	0.000 *	0.223	0.279	0.056	0.000 *	0.048	0.064	0.056	0.000 *	0.048	0.064
$(days)^2$	-0.003	0.000 *	-0.003	-0.002	-0.003	0.000 *	-0.003	-0.002	0.000	0.000 *	-0.001	0.000	0.000	0.000 *	-0.001	0.000
Asthma	0.127	0.351	-0.140	0.393	0.163	0.203	-0.088	0.413	0.272	0.006 *	0.077	0.467	0.353	0.001 *	0.152	0.554
Diabetes	0.058	0.591	-0.152	0.268	0.007	0.946	-0.203	0.218	-0.062	0.429	-0.216	0.092	-0.057	0.510	-0.226	0.112
Above-65	0.015	0.849	-0.138	0.167	0.032	0.683	-0.123	0.188	0.078	0.172	-0.034	0.189	0.069	0.281	-0.056	0.194
Poverty	-0.149	0.023 *	-0.278	-0.020	-0.136	0.040 *	-0.265	-0.006	-0.039	0.421	-0.133	0.056	-0.035	0.509	-0.139	0.069
R-squared within	0.692				0.692				0.682				0.682			
R-squared between	0.607				0.539				0.550				0.500			

© 2020 Padalabalanarayanan S et al. *JAMA Network Open.*

eTable 5. Models that Include the Interaction of SAHOs With Time Trend

**Multivariate Regression Analysis with State Random Effects Interaction of Stay-at-home and time trend State Random Effects, for Cumulative COVID-19 Cases**				
	Without FDFE (N=3023)			
Variable	*β*	*p*	*95% Confidence interval*	
Log cumulative tests	0.594	0.000*	0.536	0.652
Mask order	-0.267	0.386	-0.872	0.337
Nursing Facilities	0.052	0.364	-0.061	0.166
Total Population	0.005	0.007*	0.001	0.009
Urban population	0.042	0.000*	0.019	0.064
days	0.494	0.000*	0.451	0.537
SAHO interacted with days	-0.116	0.000*	-0.141	-0.090
$(days)^2$	-0.006	0.000*	-0.007	-0.006
SAHO interacted with $days^2$	0.002	0.000*	0.001	0.002
Asthma	0.256	0.035*	0.018	0.493
Diabetes	-0.027	0.790	-0.226	0.172
African American	0.047	0.002*	0.018	0.077
Above-65	0.049	0.516	-0.099	0.197
Poverty	-0.118	0.060	-0.241	0.005
R-squared within	0.713			
R-squared between	0.513			
rho	0.082			

© 2020 Padalabalanarayanan S et al. *JAMA Network Open.*

eFigure 1. Distribution of Percentage of Days Each State Imposed State-Level Stay at Home Orders

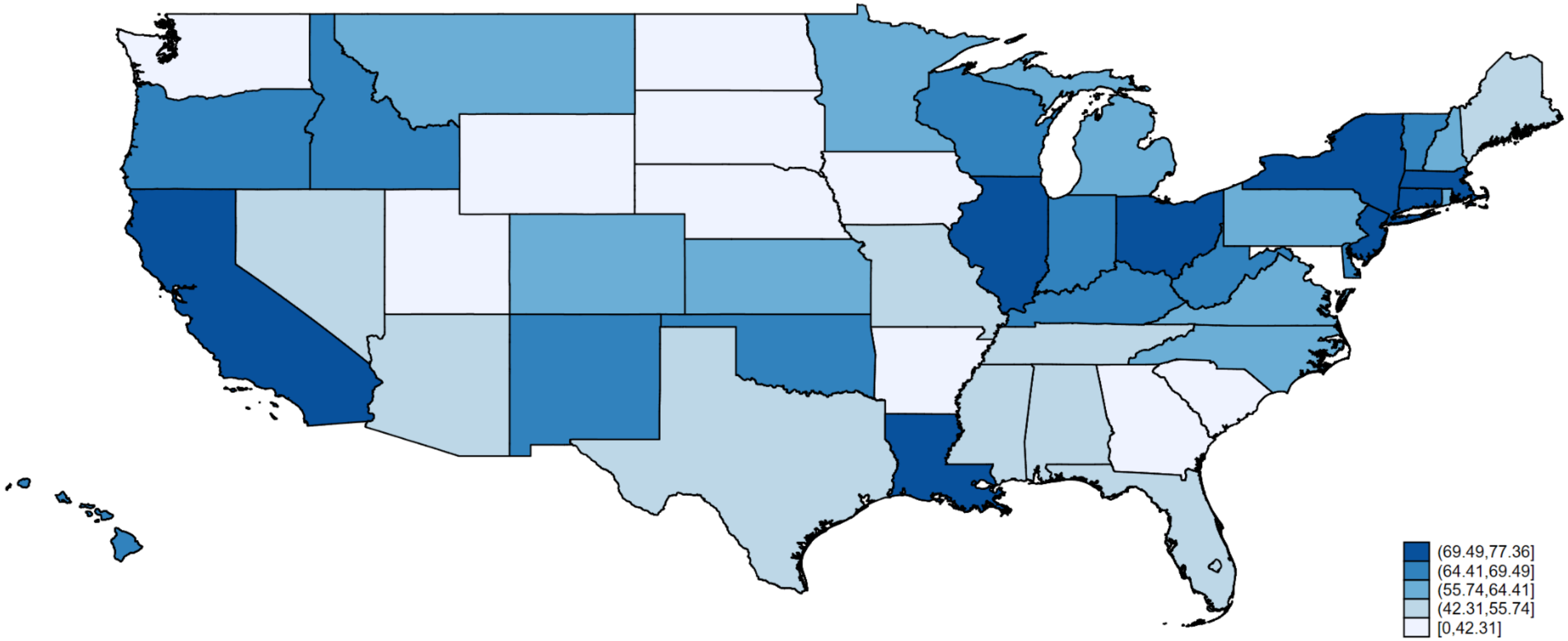

Notes: The legend represents quintiles of the distribution

© 2020 Padalabalanarayanan S et al. *JAMA Network Open.*

eFigure 2. Distribution of Percentage of African American Population in Each State

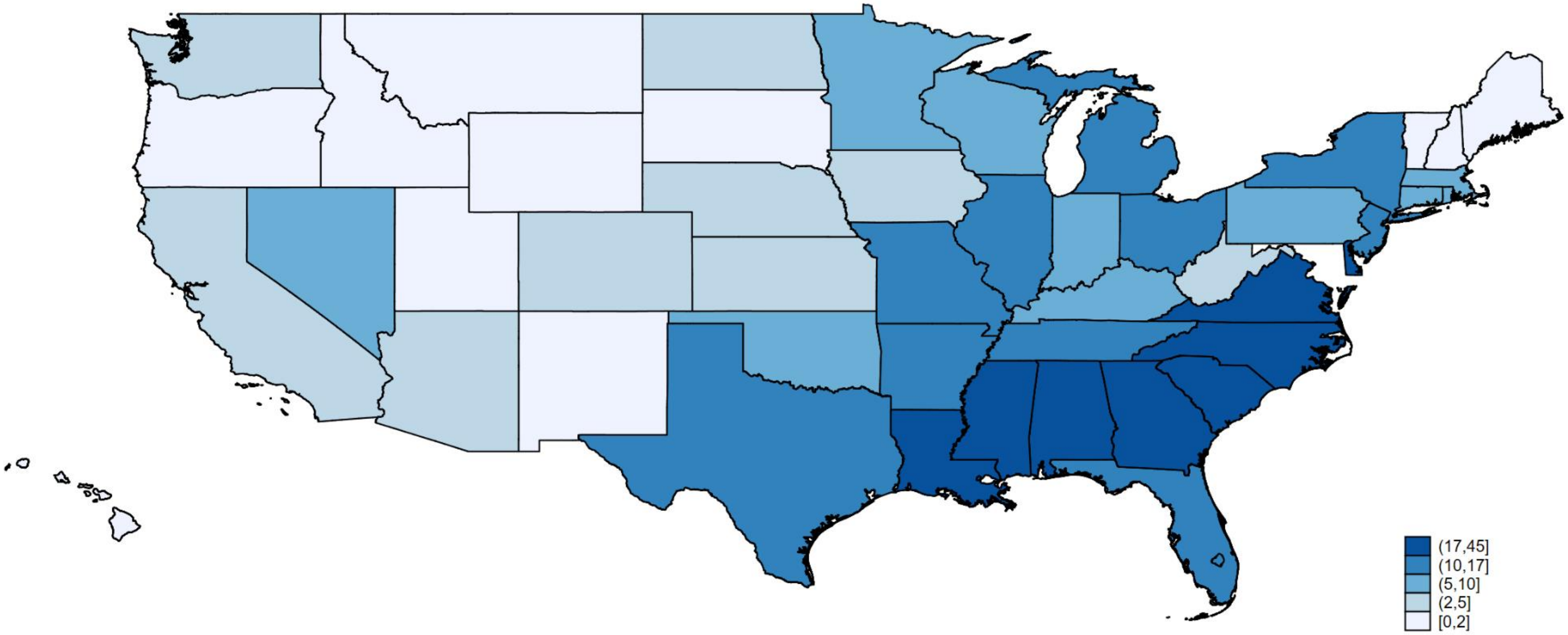

Notes: The legend represents quintiles of the distribution

© 2020 Padalabalanarayanan S et al. *JAMA Network Open.*

eFigure 3. Comparison of Reported COVID-19 Cases and Inferred Infections

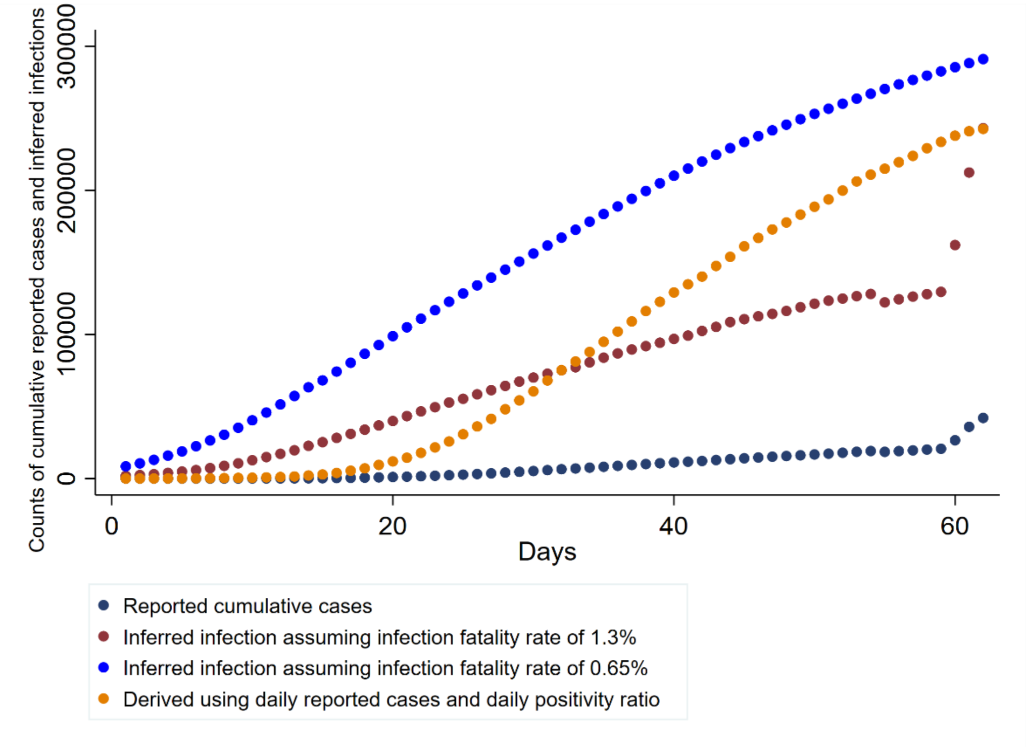

© 2020 Padalabalanarayanan S et al. *JAMA Network Open.*

eAppendix. Multivariate Regression Model With State-Specific Random Effects

$Lnoutcomes_{sd} = \beta_0 + \beta_1\ LnTestRate_{sd} + \beta_2\ Stay\text{-}Home_{sd} + \beta_3\ African\text{-}Am_s + \beta_4\ Mask\ order + \beta_5\ Nursing\ facilities + \beta 6TotPop_s + \beta_7 PoPUrban_s + \beta_8 Days_{sd} + \beta_9 (Days_{sd})^{2\,+} + \beta_{10}\ Asthma_s + \beta_{11}\ Diabetes_s + \beta_{12}\ Age65_s + \beta_{13}\ Poverty_s\ (First\_Report_s)\mu + v_s + e_{sd}.$

© 2020 Padalabalanarayanan S et al. *JAMA Network Open.*

eReferences

1. Jasmine C. Lee SM, Yuriria Avila, Barbara Harvey and Alex Leeds Matthews. See How All 50 States Are Reopening (and Closing Again). 2020; https://www.nytimes.com/interactive/2020/us/states-reopen-map-coronavirus.html. Accessed Aug 12, 2020.
2. Basu A. Estimating The Infection Fatality Rate Among Symptomatic COVID-19 Cases In The United States. 2020; https://www.healthaffairs.org/doi/10.1377/hlthaff.2020.00455. Accessed Aug 12, 2020.
3. COVID-19 Pandemic Planning Scenarios. *Centers for Disease Control and Prevention* 2020; https://www.cdc.gov/coronavirus/2019-ncov/hcp/planning-scenarios.html.
4. Gu Y. Estimating True Infections: A Simple Heuristic to Measure Implied Infection Fatality Rate. 2020; https://covid19-projections.com/estimating-true-infections/. Accessed Aug 12, 2020.

© 2020 Padalabalanarayanan S et al. *JAMA Network Open.*
